# PAI-1 in Diabetes: Pathophysiology and Role as a Therapeutic Target

**DOI:** 10.3390/ijms22063170

**Published:** 2021-03-20

**Authors:** Rawan Altalhi, Nikoletta Pechlivani, Ramzi A. Ajjan

**Affiliations:** 1 Biochemistry Department, Faculty of Science, Jeddah University, Jeddah 23235, Saudi Arabia; ml18rmqa@leeds.ac.uk; 2 Division of Cardiovascular & Diabetes Research, Leeds Institute of Cardiovascular and Metabolic Medicine (LICAMM), University of Leeds, Leeds LS2 9JT, UK; N.pechlivani@leeds.ac.uk

**Keywords:** plasminogen activator inhibitor 1 (PAI-1), PAI-1 inhibitors, diabetes, hypofibrinolysis, cardiovascular disease, therapeutics

## Abstract

Hypofibrinolysis is a key abnormality in diabetes and contributes to the adverse vascular outcome in this population. Plasminogen activator inhibitor (PAI)-1 is an important regulator of the fibrinolytic process and levels of this antifibrinolytic protein are elevated in diabetes and insulin resistant states. This review describes both the physiological and pathological role of PAI-1 in health and disease, focusing on the mechanism of action as well as protein abnormalities in vascular disease with special focus on diabetes. Attempts at inhibiting protein function, using different techniques, are also discussed including direct and indirect interference with production as well as inhibition of protein function. Developing PAI-1 inhibitors represents an alternative approach to managing hypofibrinolysis by targeting the pathological abnormality rather than current practice that relies on profound inhibition of the cellular and/or acellular arms of coagulation, and which can be associated with increased bleeding events. The review offers up-to-date knowledge on the mechanisms of action of PAI-1 together with the role of altering protein function to improve hypofirbinolysis. Developing PAI-1 inhibitors may form for the basis of future new class of antithrombotic agents that reduce vascular complications in diabetes.

## 1. Introduction

Cardiovascular disease (CVD) remains the primary cause of death in individuals with diabetes and it also results in significant morbidity, thus compromising quality of life [1]. The Framingham Heart Study has shown a 2–3-fold excess in risk of coronary artery disease (CAD), stroke, heart failure, and death from CVD among subjects with diabetes compared to individuals with normal glucose metabolism [2].

Acute vascular occlusion is usually due to the formation of an obstructive thrombus in a diseased blood vessel. Diabetes is characterised by early and more severe atherosclerosis being responsible for the high rate of vascular occlusive events in this population. Moreover, diabetes is associated with a thrombotic environment, as a result of enhanced activation of platelets and prothrombotic coagulation factors, coupled with impairment in the fibrinolytic system [3,4]. In particular, hypofibrinolysis is a key abnormality in diabetes and appears to directly contribute to the enhanced vascular risk and the adverse outcome in this population [5]. Notably, hypofibrinolysis can occur at an early age in diabetes [6] and, therefore, this abnormality warrants closer scrutiny to understand the mechanistic pathways responsible and devise more effective treatment strategies. While a number of pathways that control fibrinolysis are affected in diabetes, a central mechanism is related to alteration in plasminogen activator inhibitor (PAI)-1 levels and/or function. The current review summarises the role of PAI-1 in impaired fibrinolysis in diabetes and highlights strategies to modulate PAI-1 levels or activity as a mean to improve the fibrinolytic process and reduce thrombosis risk.

### 1.1. Fibrinolysis in Diabetes

The fibrinolytic process starts with the conversion of plasminogen into plasmin after activation by tissue-type plasminogen activator (t-PA) or urokinase-type plasminogen activator (u-PA). Plasmin is the main protein that cleaves the fibrin fibres resulting in the formation of fibrin degradation products [7]. Plasmin generation is tightly controlled not only by activators but also inhibitors to avoid excessive clot lysis. PAI-1 is one of the most powerful antifibrinolytic proteins that binds to t-PA or u-PA, inhibiting their function and reducing plasmin generation [7].

Importantly, in patients with metabolic syndrome and/or type 2 diabetes, plasma concentrations of PAI-1 are elevated, thus contributing to the hypofibrinolytic environment [8,9]. In addition to the effect on clot lysis, recent evidence suggests that increased vascular PAI-1 can directly accelerate the atherothrombotic process by promoting neointimal plaque formation [10]. This indicates that abnormalities in the coagulation system do not only affect thrombosis potential but can also contribute to the progression of atherosclerosis.

### 1.2. PAI-1 Structure and Function

#### 1.2.1. PAI-1 Structure 

**Figure 1:**
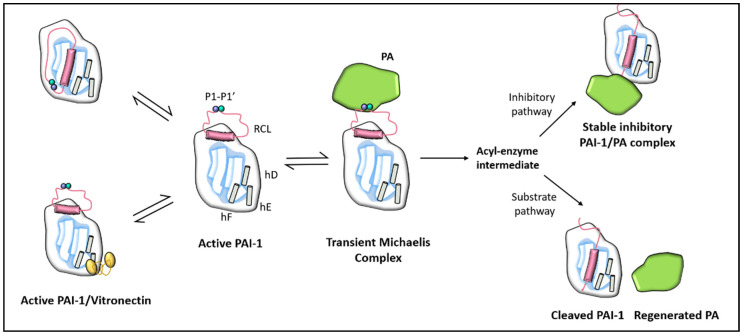
Schematic depiction of the PAI-1 conformation as well as its interaction with vitronectin cofactor and plasminogen activators (PA). PAI-1 contains two distinct interactive domains: a reactive centre loop (RCL) and a flexible joint region with helix D (hD), helix E (hE), and helix F (hF) binding sites. The P1-P1’ bond is broken to create an acyl–enzyme intermediate following the creation of a non-covalent PAI-1/PA Michaelis complex. The reaction takes place through a branched pathway, leading either to the formation of an irreversible inhibitory complex or to the generation of cleaved PAI-1 due to the intermediate acyl–enzyme hydrolysis.

PAI-1, a member of the superfamily of serine protease inhibitors (SERPIN) [11,12], is a single-chain glycoprotein of approximately 52 kDa consisting of 379 or 381 amino acids depending on heterogeneity of the N-terminal caused by two potential cleavage sites for signal peptidase [13]. PAI-1 contains two distinct interactive domains; a reactive centre loop (RCL) and a flexible joint region with helix D (hD), helix E (hE), and helix F (hF) binding sites as detailed in Figure 1 [14]. The RCL domain is the primary site for u-PA/t-PA binding and contains a P1-P1’ peptide bond that interacts with these proteases [15]. PAI-1 lacks cysteine residues and hence there is an absence of disulfide bonds that can account for its instability in solution. It includes several residues of methionine, which may explain its susceptibility to irreversible inactivation by oxidising agents.

PAI-1 exists in three structurally and functionally distinct conformations, active, latent, and cleaved (substrate) [16]. Unlike other serpins, PAI-1 is readily converted from the active to the latent state. In vitro, the conformation of active PAI-1 is spontaneously converted to an energetically more favourable inactive latent state by moving the RCL into the central β-sheet [15]. Inhibitory activity relies on the active state exposure of the RCL, so the latent form is unable to inhibit proteases. The P1-P1’ bond in the latent conformation is also inaccessible to proteolytic attack [15].

The half-life of native PAI-1 is approximately 2 h in vitro at 37 °C and is slightly longer in vivo since most of the circulating PAI-1 is in complex with vitronectin (VN) (a relatively thermostable glycoprotein capable of stabilising and transforming PAI-1 into an active form) [15,17]. It has been found that PAI-1 binds to the N-terminal ~50- amino acid somatomedin B domain of VN [18,19] and X-ray structure evidence indicates that binding to this domain induces conformational changes in PAI-1 that improves stability of the protein [15,17]. The cleaved form of PAI-1 is discussed in the next section.

#### 1.2.2. PAI-1 Function

PAI-1 is physiologically one of the most effective endogenous regulators of fibrinolysis [20]. As alluded to earlier, PAI-1 is the main inhibitor of plasminogen activation by interacting with t-PA and u-PA. This interaction involves the formation of an initial non-covalent Michaelis-like complex, followed by an acyl-enzyme intermediate and finally by the formation of an ester bond between the carboxyl group of the P1 residue of PAI-1 with the serine residue of the protease (Figure 1). With the formation of this covalent complex, the Arg^346^ and Met^347^ (P1-P1’) bond of PAI-1 is cleaved and the target protease is translocated to the opposite side of the PAI-1 molecule, thereby irreversibly inhibiting the activity of the protease [21].

### 1.3. PAI-1 Production and Regulation

#### 1.3.1. Source of PAI-1

A large number of cells have the ability to produce PAI-1 including platelets, megakaryocytes, monocytes/macrophages, adipocytes, cardiac myocytes and vascular smooth muscle cells, as well as cells of the endometrium, peritoneum, liver, mesothelium and endothelium [22]. Once synthesised, more than 90% of PAI-1 is stored in platelet α-granule, with the rest circulating in blood or deposited on the subendothelial matrix [23]. Therefore, PAI-1 levels in the blood do not reflect protein concentrations at the thrombus site given protein release from platelets when the coagulation system is activated.

PAI-1 can be differentially regulated in various tissues and is also affected by pathological conditions such as vascular disease, sepsis, inflammation, and metabolic disorders including obesity and diabetes. Since PAI-1 may show cell type-specific glycosylation and activity, the glycosylation patterns of PAI-1 may serve as potential biomarkers to predict cellular dysfunction and thrombotic risk [24].

Under physiological conditions, PAI-1 circulates in the plasma at concentrations of 10–50 ng/mL [25], rising to over 100 ng/mL in the presence of obesity, insulin resistance and diabetes [24]. There is a circadian pattern to PAI-1 plasma levels, which peak in the early morning corresponding to a nadir in fibrinolytic activity, while they fall in the afternoon [25,26]. This seems to be related to stimulation of PAI-1 production by cortisol, which peaks in the early morning and clinically may contribute to the increased risk of myocardial infarction at this time of the day. Moreover, PAI-1 plasma levels vary according to race/ethnicity [27,28] and gender [29], although variations in body structure and distribution of adipose tissue can account for much of this variability.

#### 1.3.2. Regulation of PAI-1

**Figure 2:**
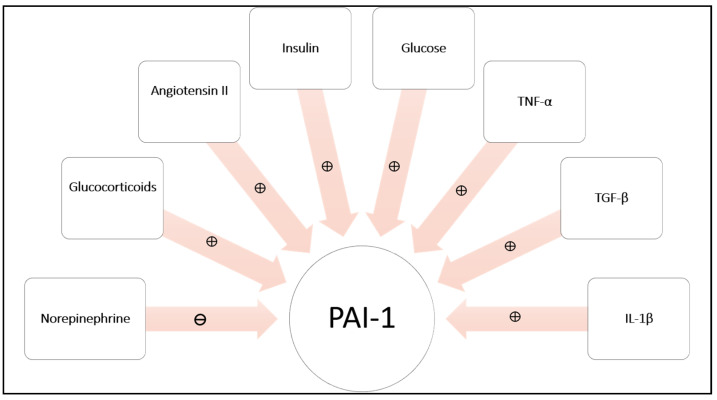
Adipocyte expression and secretion of plasminogen activator inhibitor-1 (PAI-1) is stimulated by glucocorticoids, angiotensin II (Ang II), insulin, glucose and a variety of cytokines, including tumour necrosis factor-α (TNF-α), transforming growth factor-β (TGF-β), and interleukin-1β (IL-1β). Catecholamines, such as norepinephrine inhibit production of PAI-1.

A variety of factors regulate the expression of PAI-1 (Figure 2), including glucocorticoids [30], insulin, glucose and inflammatory cytokines [31]. It is worth noting that the expression of transforming growth factor-β (TGF-β), tumour necrosis factor-α (TNF-α), and interleukin-1β (IL-1β) is upregulated in adipocytes of obese individuals and those with diabetes [32], providing a mechanism for increased PAI-1 levels in obesity and insulin resistant states. The synthesis of PAI-1 is also increased by angiotensin II (Ang II), which acts via type 1 receptor of angiotensin II expressed in adipocytes [33]. In contrast, catecholamines down-regulate PAI-1 gene expression [9]. 

### 1.4. Role of PAI-1 in Diabetes 

Plasma levels of PAI-1 are elevated in individuals with type 2 diabetes, although this does not necessarily hold true for type 1 diabetes (Table 1) [34]. These findings suggest the main mechanism for elevated PAI-1 levels in diabetes is related to obesity and insulin resistance, rather than elevated glucose levels [35]. However, hyperglycaemia may still have an effect in the presence of insulin resistance. A role for PAI-1 in vascular pathology is supported by protein accumulation at vascular atheroma sites, which appear to be particularly pronounced in those with diabetes [25].

Insulin was found to increase PAI-1 expression in a HepG2 hepatocyte cell line [36]. The combination of insulin and insulin-like growth factor 1 further demonstrated a synergistic effect on PAI-1 expression [37]. Moreover, insulin precursors, both proinsulin and split products of proinsulin, also enhanced PAI-1 expression and levels of these proteins are known to be elevated in type 2 diabetes (T2D) [38]. Therefore, insulin resistance, a hallmark of T2D, increases PAI-1 expression secondary to increased levels of insulin and its precursors. In addition to increased PAI-1 mRNA expression, insulin may also decrease the rate of mRNA degradation thus resulting in sustained protein production [31].

Insulin resistance causes a disruption in the phosphoinositide-3 kinase (PI3-K/Akt) signalling pathway, which results in insufficient tissue insulin sensitivity. During compensatory hyperinsulinemia, the paradox of pathologies in molecular insulin signalling leads to decreased activity of the PI3-K/Akt pathway together with upregulation of the mitogen-activated protein kinase/extracellular signal-regulated kinase (MAPK/ERK) pathway [39]. Insulin resistance is linked to glycotoxicity, lipotoxicity, and inflammation, which contribute to the initiation and progression of atherogenesis and vascular disease [40,41]. Insulin resistance and endothelial dysfunction are linked by changes in the balance between the PI3-K/Akt and MAPK/ERK pathways, explaining the high risk of atherothrombosis in insulin-resistant states [42]. Furthermore, when the balance of insulin resistance is transferred towards the MAPK/ERK pathway, insulin releases inflammatory markers such as PAI-1, intercellular adhesion molecule 1 (ICAM-1), vascular cell adhesion molecule 1 (VCAM-1), and E-selectin, which leads to endothelial dysfunction, which in turn predisposes to vascular pathology [43].

Hyperglycemia and hypertriglyceridemia are metabolic disorders caused by insulin deficiency, either relative or absolute. In vitro, increased glucose concentrations enhance PAI-1 expression in both endothelial and vascular smooth muscle (VSM) cells [44]. In HepG2 cells, triglycerides and their constituents (fatty acids) increase PAI-1 expression [45]. Furthermore, the combination of elevated insulin and triglyceride levels induces a synergistic increase in PAI-1 production [46].

Insulin resistance is also associated with lower very low-density lipoprotein (VLDL) clearance [47]. By inducing transcription of the PAI-1 gene promoter, VLDL particles have been shown to increase PAI-1 biosynthesis in endothelial cells while also stabilising the mRNA transcripts of the protein. The main signalling pathway involved in VLDL-induced PAI-1 biosynthesis is related to MAPK activation [48]. Therefore, a combination of factors, related to hyperglycemia and insulin resistance, are responsible for increased PAI-1 production in T2D, through well-defined pathways.

**Figure 3:**
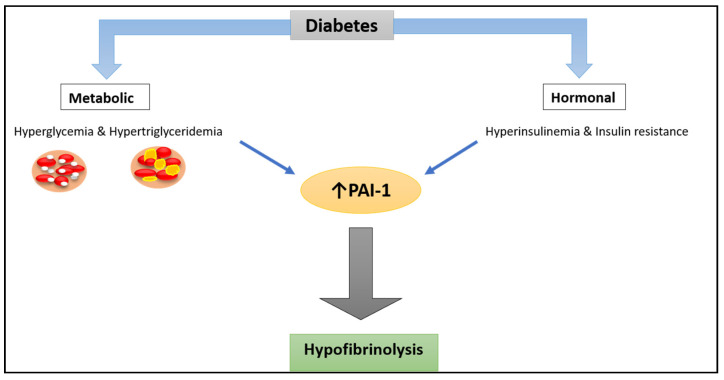
Mechanism of increased PAI-1 levels in diabetes. Hormonal (hyperinsulinemia) and metabolic (hyperglycemia and hypertriglyceridemia) derangements in T2D patients, seem to have a role in elevating PAI-1 levels in this population, resulting in hypofibrinolysis that causes pathological deposition of fibrin and damage to the tissues.

Moreover, studies show higher plasma PAI-1 levels in individuals with diabetes and recent myocardial infarction (MI) compared to those without diabetes. Increased activity of PAI-1 has been implicated in impaired pharmacological and spontaneous reperfusion after acute MI, directly linking abnormalities in this protein to adverse outcomes in these patients [25]. Metabolic and hormonal mechanisms operating to alter PAI-1 levels in diabetes are depicted in Figure 3, while Table 1 summarises key studies investigating PAI-1 levels in individuals with type 2 diabetes.

### 1.5. Role of PAI-1 in CVD

Prospective studies have shown that impaired fibrinolysis and specifically elevated PAI-1 concentrations are independent risk factors for both arterial and venous thrombotic occlusive vascular disease [30,58]. Moreover, it is suggested that patients with elevated plasma PAI-1 have the highest risk of early re-infarction [59] and therefore protein levels have prognostic significance following cardiac ischaemia. Importantly, a positive association between PAI-1 activity of fathers with premature MI and their children has recently been reported and therefore PAI-1 levels can also have a prognostic role in familial predisposition to CVD [23,60].

### 1.6. Role of PAI-1 in Diabetic Retinopathy and Chronic Kidney Disease

A number of studies have documented an association between PAI-1 and microvascular complications such as retinopathy and chronic kidney disease. One study suggested that elevated PAI-1 plasma levels are independently associated with lower risk of retinopathy [61]. However, a much larger longitudinal study involving 858 individuals from the Veterans Affairs Diabetes Trial (VADT) cohort has shown that PAI-1 plasma levels predict the development of retinopathy in T2D with a 12% increased risk for every 10 ng/dl increase in PAI-1 levels [62]. Moreover, PAI-1 levels were elevated in 153 Chinese diabetes patients with advanced diabetic retinopathy, suggesting an association with disease severity [63]. Interestingly, animal work has shown increased PAI-1 expression in the retina with the development of proliferative changes, directly implicating PAI-1 in disease pathogenesis [64]. Taken together, studies generally agree that elevated PAI-1 levels carry a higher risk of retinopathy, although the exact mechanisms remain an area for future research.

A number of studies have also reported a relationship between elevated plasma PAI-1 levels and chronic kidney disease (CKD). Many factors can induce PAI-1 expression in CKD, such as TGF-β and angiotensin-II. The most compelling evidence for a role of PAI-1 in renal disease is derived from prevention of CKD, and even disease regression, with reduction in PAI-1 production in animal models [65,66]. Moreover, data investigating the effects of two PAI-1 inhibitors on kidney injury in diabetic mice suggested that this approach improved kidney function [67]. Another study on PAI-1 deficient mice demonstrated that PAI-1 directly regulates TGF-β expression by binding to u-PAR and activating the extracellular-regulated signal kinase (ERK)/MAPK pathway thus contributing to renal disease [68].

## 2. Targeting PAI-1 for Potential Therapies

**Figure 4:**
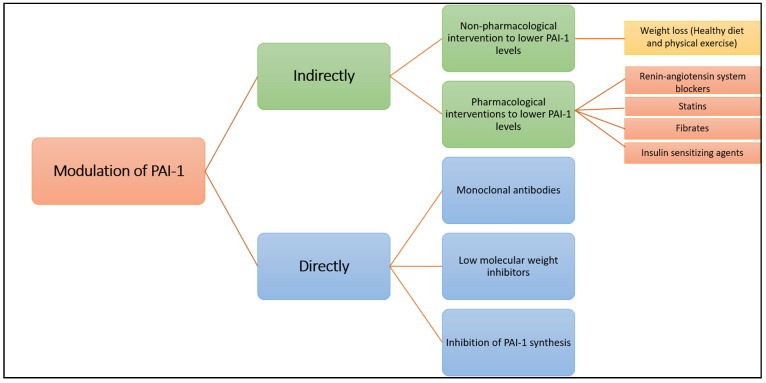
Summary of the strategies for modulating PAI-1 indirectly and directly.

Since elevated PAI-1 plasma levels and activity contribute to cardiovascular pathology, several groups attempted to use PAI-1 as a therapeutic target in order to improve plasmin generation and alleviate the hypofibrinolytic environment [23]. We provide here an overview of the different approaches used to modulate PAI-1 production or function (summarised in Figure 4).

### 2.1. Indirect Modulation of PAI-1 Production or Function

#### 2.1.1. A. Non-Pharmacological Intervention to Lower PAI-1 Levels

##### Weight Loss (Healthy Diet and Physical Exercise)

Weight loss is one of the best methods to improve obesity-related fibrinolytic impairment [9], partly related to a marked decrease in PAI-1 production, which has been demonstrated in a number of studies [69]. It has also been shown that not only weight loss but also increased physical activity are linked to decreased PAI-1 levels [22]. Importantly, the decrease in PAI-1 levels is associated with improved clinical parameters; Killewich et al. [70] found that patients with intermittent claudication, due to peripheral artery disease, had improvement in their symptoms after a programme of physical exercise, which was associated with decreased PAI-1 activity and enhanced fibrinolytic activity. Interestingly, those with higher baseline PAI-1 levels gained the most clinical benefit from the intervention, directly implicating protein levels in disease presentation [71].

#### 2.1.2. Pharmacological Interventions to Lower PAI-1 Levels

##### Renin-Angiotensin System Blockers

The activation of the renin–angiotensin system is closely related to PAI-1 [72]. Angiotensin II (also produced by adipose tissue) is a biologically active angiotensinogen processing agent and has been shown to stimulate PAI-1 expression at the transcriptional level in human adipocytes [33]. The effects of angiotensin-converting enzyme (ACE) inhibitors on cardio protection appear to be mediated by pathways that are partially independent of the reduction in blood pressure. Vaughan et al. reported that one proposed mechanism is related to modulation of PAI-1 concentrations [73]. However, others failed to show an effect of ACE inhibitors on PAI-1 levels [74]. Therefore, this remains an unresolved area and future work is required to understand the role of ACE inhibitors on fibrinolysis in general and PAI-1 activity/levels in particular [75].

##### Statins

Anti-hyperlipidaemic agents, in particular statins, have been shown to modulate the prothrombotic environment and affect the fibrinolytic system [76]. Statins inhibit the expression of PAI-1 and their use is associated with reduction in PAI-1 levels, which may explain improved t-PA activity in those treated with these agents [16,77,78].

##### Fibrates

Fibrates can decrease the in vitro production of PAI-1 independently of the triglyceride reducing effect [79]. In vivo use of fibrates also decreases PAI-1 levels and reduces protein expression in human arterial smooth muscle cells (SMC) [79,80]. Different fibrates showed different potencies to suppress PAI-1 synthesis, but these effects are generally mild and the clinical role of these observations remain unclear [81].

##### Insulin-Sensitising Agents

Insulin-sensitising agents, such as metformin and thiazolidinediones have been shown to have beneficial effects on the fibrinolytic system, mediated, at least in part, by a reduction in PAI-1 levels [82]. Glucagon-like peptide-1 receptor agonists inhibit TNF-α stimulated PAI-1 production in vitro [83], while metformin reduces PAI-1 levels in vivo, an effect believed to be related to enhanced insulin sensitivity and/or reduction in proinsulin production [8,84,85].

### 2.2. Direct Modulation of PAI-1 Production or Activity

**Figure 5:**
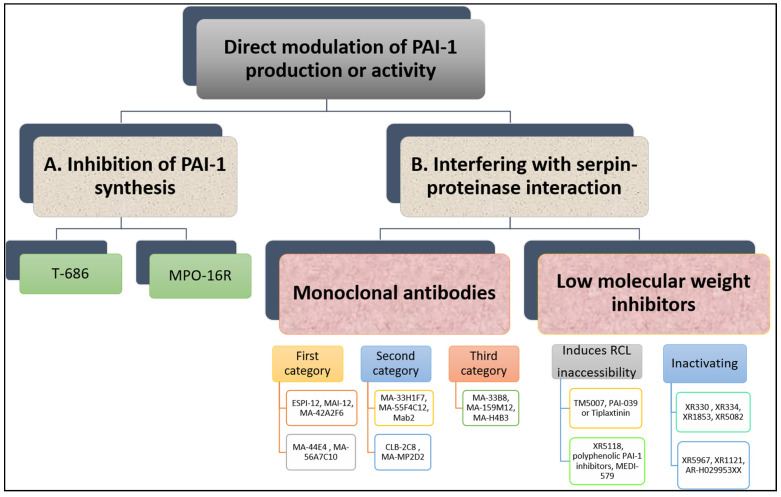
Overview of direct modulation of PAI-1 production or activity by (A) inhibition of PAI-1 synthesis or (B) interfering with serpin–proteinase interaction.

Many research groups have invested in the development of PAI-1 inhibitors over the past three decades [86] either through inhibiting PAI-1 synthesis or interfering with serpin-proteinase interaction (Figure 5). Despite large volume of research in this area, no PAI-1 inhibitor is yet available for routine clinical use.

#### 2.2.1. Inhibition of PAI-1 Synthesis

Reduction in PAI-1 synthesis represents an attractive approach to enhancing fibrinolysis and reducing thrombosis risk. However, this needs to be undertaken in a controlled manner as full inhibition of PAI-1 synthesis predisposes to mild-severe bleeding events, supported by evidence from individuals with complete PAI-1 deficiency [87]. Therefore, ideally agents suppressing PAI-1 production should reduce excess PAI-1 levels to attain normal levels and restore normal haemostasis [88].

In vitro studies have shown that TGF-β-stimulated PAI-1 production in cultured endothelial cells is attenuated by a butadiene derivative, T-686. Furthermore, T-686 also diminished the rise in PAI-1 activity in aortic atherosclerotic lesions of rabbits. This was accompanied by decreased aortic PAI-1 mRNA expression, associated with a reduction in the development of atherosclerotic lesions [89]. Pawlowska et al. [90] showed that an oligonucleotide antisense to PAI-1 mRNA (MPO-16R) decreased PAI-1 concentration in rat plasma and platelets, while MPO-16R induced a large delay in occlusion time in an experimental model of rat arterial thrombosis. Therefore, reducing PAI-1 production is possible and preliminary evidence suggests this strategy can alter the atherothrombotic process. However, findings from these studies have not been translated into work in man and the clinical future of this antithrombotic strategy is uncertain.

#### 2.2.2. Interfering with Serpin-Proteinase Interaction

Although the available evidence is very preliminary, studies have shown that inhibition of PAI-1 enhances endogenous fibrinolytic activity without directly affecting blood coagulation or platelet function [91]. Several PAI-1 inhibitors have been studied in vitro and in vivo and have shown variable antithrombotic efficacy.

##### Monoclonal Antibodies

A variety of monoclonal antibodies against human PAI-1 has been developed to modulate protein activity [11]. These can be subdivided into at least three different categories. First, monoclonal antibodies may prevent the formation of the initial Michaelis complex formed between PAI-1 and its target protease. It was found that monoclonal antibodies against PAI-1, including ESPI-12, MAI-12, MA-42A2F6, MA-44E4 and MA-56A7C10, with their epitopes located near the RCL of PAI-1, interfere directly with the PAI-1/protease interaction [92]. MAI-12, one of the PAI-1 binding antibodies that inhibit complex formation with the target, has been studied extensively in vitro and in an in vivo venous thrombosis model [93]. MAI-12 inhibited human and rabbit PAI-1 in vitro and significantly enhanced endogenous thrombolysis in a rabbit model of jugular vein thrombosis and partially prevented thrombus extension [93].

A second category of PAI-1 inhibiting antibodies are the so-called “switching antibodies”, which inhibit PAI-1 activity by cleaving and inactivating the protein. These antibodies include MA-33H1F7, MA-55F4C12 [94], Mab2 [95], CLB-2C8 [96] and MA-MP2D2 [97]. The murine monoclonal antihuman PAI-1 antibody CLB-2C8 showed great promise as it inhibited protein activity in different species [98], while in vivo administration significantly enhanced endogenous thrombolysis, and reduced thrombus growth in a rabbit jugular vein thrombosis model [98]. Despite the early promise, CLB-2C8 was not followed up with human studies for reasons that are unclear.

As for the third category of PAI-1 inhibiting antibodies, these are known for accelerating conversion of active PAI-1 to its latent conformation and include MA-H4B3, MA-159M12 and MA-33B8 antibodies [99]. The monoclonal antibody, MA-33B8, was reported to convert PAI-1 to latent conformation and random mutagenesis was used to identify the MA-33B8 epitope in the PAI-1 molecule to comprehend the mechanism of action [100]. Surface plasmon resonance showed 100-fold higher affinity of MA-33B8 to latent PAI-1 than active protein [100]. Additionally, structural modelling results specified the presence of a particular intermediate structure of PAI-1 that is stabilised by MA-33B8 binding [100]. It was suggested that this intermediate form of PAI-1 has a partial insertion into β-sheet A of the reactive centre loop, supporting the hypothesis that active serpins have flexible RCLs, and that this flexibility is crucial for the role of the inhibitor [101]. As a result, the activity of the serpin may also be blocked by synthetic peptides homologous to the RCL serpin converting PAI-1 to a shape similar to the latent form [102]. Moreover, Ngo et al. [103] reported that antibody MA-159M12 promoted the conversion of active to latent conformation, further indicating this is a viable approach to alter PAI-1 activity. Antibody MA-H4B3 has been shown to inactivate recombinant PAI-1 in a time-dependent way [104], which indicates that, in the presence of MA-H4B3, incorporation of the RCL is accelerated, suggesting that the loss of activity is the result of the transition of latency [104].

##### Low Molecular Weight Inhibitors

Several PAI-1 low molecular weight antagonists have been discovered and characterised. The first were diketopiperazines (XR330 and XR334) and, from that template, more potent antagonists were designed (XR1853, XR5082, XR5967, XR1121 and XR5118) which inhibit PAI-1 by inducing the transition from active PAI-1 to non-reactive PAI-1 [105,106]. XR334, XR1853 and XR5082 effectively increased fibrinolysis in vivo by inhibiting the interaction of t-PA/u-PA and PAI-1 in a rat carotid artery thrombosis model [107].

XR5118 administration improved ex vivo fibrinolysis and protected against thrombus formation in a rat electrically stimulated carotid artery (ESCA) model [23,108]. The protection in this arterial thrombosis was associated with a significant decrease in PAI-1 activity and a significant increase in t-PA activity in plasma [23,108]. Importantly, inhibition of PAI-1 by XR5118 did not contribute to any increase in rat bleeding time, which held promise for the safety of this compound [108].

Furthermore, TM5007, a derivative of indole oxoacetic acid was shown to effectively inhibit PAI-1 activity [91]. This compound is metabolically stable, non-toxic, and has demonstrated strong oral bioavailability and in vivo efficacy in a rat thrombosis model [91].

Björquist et al. [109] developed the flufenamic acid-based AR-H029953XX after it was reported that flufenamic acid and its derivatives enhanced in vitro plasma clot lysis in an assay of plasmin generation [110].

As for PAI-039 or Tiplaxtinin, an indole derivative, seemed a very promising PAI-1 inhibitor [111]. It has been examined in several animal models confirming a putative value of PAI-1 inhibition for therapeutic purposes [112]. In another study, however, Tiplaxtinin did not inhibit the activity of PAI-1 in rat plasma 15 min after intravenous injection, which was suggested to be due to the fact that Tiplaxtinin is unable to inhibit vitronectin bound PAI-1 because of the overlapping epitopes of vitronectin and Tiplaxtinin on PAI-1 [113].

A novel class of polyphenolic PAI-1 inhibitors was identified by Cale et al. [114], representing an enhanced 10–1000-fold potency over previously described PAI-1 inhibitors. This class exerted its PAI-1-inhibitor effect by blocking the initial bond between the protein and protease. These compounds inactivate PAI-1 in the presence of vitronectin, giving them an advantage over other PAI-1 inhibitors. Two of these compounds demonstrated efficacy in plasma and one other blocked PAI-1 activity in in vivo mice studies. The authors proposed that the known cardiovascular advantages of dietary polyphenols may be due in part to PAI-1 inactivation, although this is only a hypothesis that is yet to be tested. A human antibody, MEDI-579, has recently been shown to bind to the active form of human PAI-1 with high affinity and specificity [115]. Importantly, MEDI-579 effects were still evident in vitronectin bound PAI-1. Crystallography demonstrated that this specificity is achieved due to the attachment of MEDI-579 Fab to the RCL of PAI-1 and to the same exosite used by both t-PA and u-PA [115]. This indicates that MEDI-579 is able to modulate the interaction of PAI-1 with t-PA and u-PA in a manner not previously defined for a human PAI-1 inhibitor by directly competing with proteases for RCL binding.

## 3. Conclusions

People with diabetes suffer from premature atherothrombosis, resulting in a high rate of morbidity and mortality. Hypofibrinolysis contributes to the risk of vascular events and is partly mediated by elevated levels/activity of PAI-1. Therefore, the development of agents that modulate the hypofibrinolytic milieu in diabetes is one alternative strategy to reduce the risk of vascular thrombosis in this population.

Research over the past few decades has shown that PAI-1 is a complex protein with different conformations and associations with other proteins, which can affect its function. This contributed to the difficulties in identifying a reliable agent that inhibits protein function to ameliorate the hypofibrinolytic environment. Different strategies have been explored over the years to reduce PAI-1 production and/or modulate protein activity. A large number of compounds have been identified, ranging from chemicals, monoclonal antibodies to small molecules. Despite favourable in vitro and animal in vivo effects for some of these agents, none made it into human studies for reasons that are not entirely clear. The suspicion is that human ex vivo work was disappointing, or the various compounds were found to have “off target” or toxic effects preventing human use. This highlights the importance of publishing “negative studies” in order to make the scientific community better informed and avoid duplicating work that has already been undertaken.

The newer and more refined PAI-1 inhibitors offer the promise of having effective agents that modulate hypofibrinolysis and reduce clinical thrombotic events while potentially limiting bleeding risk; the quest to find an effective and safe PAI-1 inhibitor in man continues.

## Figures and Tables

**Table 1:** Summary of main studies investigating PAI-1 levels in individuals with type 2 diabetes (T2D). Values are presented as mean ± SD or median interquartile range (IQR). Variation in PAI-1 levels is likely due to the different methodologies used to measure protein levels and potential differences between study populations.

Study	Country	No. of T2D/Controls	Mean ± SD Median (IQR) of T2D	Mean ± SD Median (IQR) of Control	*p*-Value
Kitagawa (2006) [49]	Japan	47/31	82.7 ± 54.5 ng/mL	52.9 ± 51.7 ng/mL	<0.05
Soares (2010) [50]	Brazil	25/12	108.8 ± 48.5 ng/mL	37.6 ± 33.2 ng/mL	<0.05
Le (2008) [51]	U.S.A.	104/59	39(24–59) ng/mL	31(18–53) ng/mL	NS
Romuk (2008) [52]	Poland	20/21	10.57 ± 5.8 IU/mL	3.90 ± 1.7 IU/mL	<0.0001
Sahli (2009) [53]	Sweden	69/80	39.7 ± 35.3 IU/mL	10.5 ± 12.3 IU/mL	<0.0001
Erem (2005) [54]	Turkey	92/40	44.6 ± 10.6 ng/mL	21.4 ± 5.8 ng/mL	<0.0001
Verkleij (2011) [55]	Netherlands	207/100	98 ± 102 ng/mL	57 ± 38 ng/mL	0.038
Krekora (1997) [56]	Italy	59/50	107 ± 28 ng/mL	29.1 ± 17 ng/mL	<0.001
Hernandez (2000) [57]	Spain	41/40	51.3 ± 29 ng/mL	23 ± 16.2ng/mL	<0.001
